# Identifying Neisseria Species by Use of the 50S Ribosomal Protein L6 (*rplF*) Gene

**DOI:** 10.1128/JCM.03529-13

**Published:** 2014-05

**Authors:** Julia S. Bennett, Eleanor R. Watkins, Keith A. Jolley, Odile B. Harrison, Martin C. J. Maiden

**Affiliations:** Department of Zoology, University of Oxford, Oxford, United Kingdom

## Abstract

The comparison of 16S rRNA gene sequences is widely used to differentiate bacteria; however, this gene can lack resolution among closely related but distinct members of the same genus. This is a problem in clinical situations in those genera, such as Neisseria, where some species are associated with disease while others are not. Here, we identified and validated an alternative genetic target common to all Neisseria species which can be readily sequenced to provide an assay that rapidly and accurately discriminates among members of the genus. Ribosomal multilocus sequence typing (rMLST) using ribosomal protein genes has been shown to unambiguously identify these bacteria. The PubMLST Neisseria database (http://pubmlst.org/neisseria/) was queried to extract the 53 ribosomal protein gene sequences from 44 genomes from diverse species. Phylogenies reconstructed from these genes were examined, and a single 413-bp fragment of the 50S ribosomal protein L6 (*rplF*) gene was identified which produced a phylogeny that was congruent with the phylogeny reconstructed from concatenated ribosomal protein genes. Primers that enabled the amplification and direct sequencing of the *rplF* gene fragment were designed to validate the assay *in vitro* and *in silico*. Allele sequences were defined for the gene fragment, associated with particular species names, and stored on the PubMLST Neisseria database, providing a curated electronic resource. This approach provides an alternative to 16S rRNA gene sequencing, which can be readily replicated for other organisms for which more resolution is required, and it has potential applications in high-resolution metagenomic studies.

## INTRODUCTION

Rapid and reliable identification of bacteria is fundamental to experimental microbiology, particularly in clinical settings where it is frequently necessary to distinguish organisms which are genetically very closely related but which have stable and distinct disease phenotypes. A good example is the genus Neisseria, which comprises mostly commensal inhabitants of the mucosal surfaces of humans and animals but includes two significant pathogens, Neisseria gonorrhoeae, the gonococcus, which causes gonorrhea, and Neisseria meningitidis, the meningococcus, which can cause meningitis and septicemia. As the meningococcus is an “accidental pathogen,” which is frequently carried but rarely invasive, species identification is particularly important in community studies, where meningococcal carriage rates are estimated in the presence of related species which are not easily distinguished using conventional methods. This is especially important when vaccines are being introduced, such as the recently developed protein-polysaccharide conjugate serogroup A vaccine (PsA-TT; MenAfriVac) (1). Conventional phenotypic identification of bacteria is time-consuming and difficult to deploy, especially in resource-limited settings, and may suffer from errors in interpretation leading to misidentification.

For isolate characterization purposes, approaches based on DNA sequencing offer accuracy and reproducibility with the additional advantage that the data generated can be transferred electronically and stored on public databases. For many years, sequence analysis of 16S rDNA, encoding 16S rRNA (ribosomal DNA sequencing), has played a principal role in this endeavor. In this approach, part or all of the 16S rRNA gene is sequenced, and identification is achieved by comparison of this sequence to curated sequences on web-accessible databases (for example http://www.ridom.de/rdna/ [2] and http://eztaxon-e.ezbiocloud.net/ [3]). The 16S rRNA gene has been a valuable target as it is ubiquitous and composed of both conserved and variable regions, allowing the design of universal PCR primers to generate nucleotide sequences that can be used to differentiate among isolates. The 16S rRNA molecule is so conserved, however, that very similar or identical sequences are frequently present in more than one species which have distinct and stable phenotypic properties (4–6).

Recently, ribosomal multilocus sequence typing (rMLST) (7) has been proposed as a method which provides an additional rational and universal approach to species classification. This approach exploits the availability of whole-genome sequence (WGS) data by indexing variation at the 53 genetic loci encoding the bacterial ribosomal protein genes. This method has been shown to unambiguously determine the species identity of Neisseria isolates, demonstrating good congruence with both whole-genome analyses and more conventional approaches (4). These data indicated that some species had been misidentified using conventional methods, and that minor changes in nomenclature were required (8). The rMLST approach, however, requires nucleotide sequence variation data at 53 loci and, although these are readily extracted from WGS data, such information is not always economically or practically available from all specimens. Therefore, the loci in the rMLST scheme were examined to identify a gene fragment from a single locus that can be used to rapidly identify Neisseria species in both the diagnostic and research settings. The target identified, a 413-bp fragment of the 50S ribosomal protein L6 (*rplF*) gene, includes both conserved regions suitable for primer design and variable regions to distinguish sequences from different Neisseria species. Comparison of the *rplF* gene fragments provided sufficient discrimination to identify most species within the genus accurately, rapidly, and inexpensively.

## MATERIALS AND METHODS

### Isolates and genome sequences.

Nucleotide sequences were obtained from 44 genomes which were part of the data set used to validate rMLST in Neisseria (4); a different set of 44 Neisseria DNA samples (a gift from Bachra Rokbi, Sanofi Pasteur, Marcy l'Etoile, France), which were used to validate the assay using Sanger sequencing (see Table S1 in the supplemental material); and 839 publicly available genome sequences downloaded from the PubMLST Neisseria database (http://pubmlst.org/neisseria/) (9), including those deposited as part of the MRF Meningococcus Genome Library (www.meningitis.org/research/genome). All isolates analyzed are listed in Table S2 in the supplemental material, including culture collection isolates and the type strains of Neisseria polysaccharea, Neisseria cinerea, Neisseria lactamica, Neisseria subflava, Neisseria mucosa, Neisseria oralis, Neisseria weaveri, Neisseria bacilliformis, Neisseria dentiae, Neisseria shayeganii, Neisseria canis, Neisseria wadsworthii, Neisseria animalis, and Neisseria elongata and the type strains of the previous species, Neisseria sicca, Neisseria macacae, and Neisseria flavescens (8).

### Extracting and analyzing sequence data from the PubMLST Neisseria database.

Nucleotide sequences from the 53 concatenated ribosomal protein genes used in rMLST, the seven housekeeping gene fragments used in MLST, individual ribosomal protein genes, and the 16S rRNA gene were extracted from the PubMLST Neisseria database (9). Individual allele designations were also extracted from the database. Sequences were aligned with Muscle version 3.7 (10), and Mega5 (11) was employed to reconstruct phylogenies using the neighbor-joining method. Genetic distances were determined according to the Kimura two-parameter model (12), with all ambiguous positions removed from each pairwise sequence comparison and bootstrap values (13) based on 1,000 replications. DNA divergence between sequences was calculated using DnaSP5 (14), with fixed nucleotide sequence differences defined as sites at which all of the sequences in one sample are different from all the sequences in a second sample.

### Nucleotide sequence determination.

The *rplF* fragment was amplified using the PCR primers *rplF*-F (5′-CAGTGACTGTTCCCGCTGGTGT-3′) and *rplF*-R (5′-AGGYTCAGGAGKWCGGAAHG-3′), which were designed using the primer-BLAST tool (15) available from the National Center for Biotechnology Information (http://www.ncbi.nlm.nih.gov/) and MEGA5 (11). For PCR amplification of the *rplF* gene fragment, reaction mixes were incubated for 35 cycles; each cycle consisted of 95°C for 30 s, 55°C for 30 s, and 72°C for 1 min. PCR products were purified using a precipitation method (16) and the nucleotide sequences of the purified PCR products were determined on each DNA strand using the primers described above by cycle sequencing with Applied Biosystems BigDye ready reaction mix (Life Technologies), used in accordance with the manufacturer's instructions. Sequence termination reaction products were separated and the sequence data collected using an Applied Biosystems 3730 DNA analyzer (Life Technologies). Nucleotide sequence data from forward- and reverse-strand electropherograms were assembled into single contiguous sequences using SeqSphere (http://www.ridom.de/seqsphere/) and checked using the Staden suite of programs (17).

### Defining *rplF* fragment alleles and associating with species.

The database was seeded with the first *rplF* fragment allele identified (arbitrarily assigned allele 1), and all genomes in the PubMLST Neisseria database were searched against this sequence (scanned) for the *rplF* fragment allele within the BIGSdb software using the BLAST algorithm (18). All variants with distinct nucleotide sequences were assigned unique allele designations. Each allele was also assigned a genospecies association, based on rMLST species designations (4), with type strains used to confirm these designations, where available. If genome sequences for type strains were unavailable, seven locus MLST data were used to confirm species identity (19). A reference table of alleles with associated genospecies was constructed within the PubMLST Neisseria database, which can be used to compare *rplF* fragment sequences to aid species identification. If an allele was obtained from a type strain or had an associated rMLST profile, the genospecies was considered confirmed; if not, it was considered provisional and labeled as such within the database.

## RESULTS

### Phylogenetic analysis of ribosomal protein genes.

**FIG 1:**
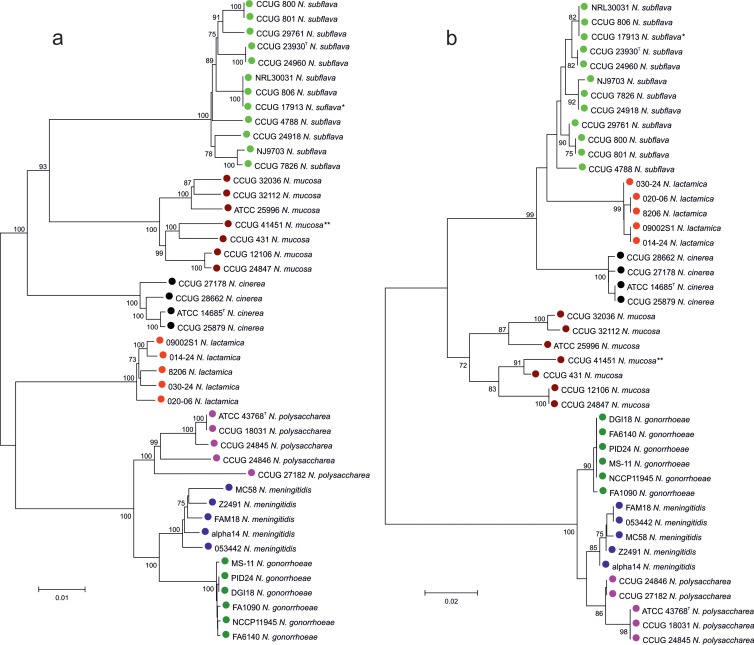
Evolutionary relationships among 44 Neisseria species based on concatenated sequences from 53 whole ribosomal protein genes and single *rplF* gene fragments. (a) Concatenated sequences from 53 ribosomal protein genes. (b) *rplF* gene fragments. Type strains of previous species: *, *N. flavescens*; **, N. macacae. The percentages of replicate trees in which the associated taxa clustered together in the bootstrap test (1,000 replicates) are shown next to the branches. The unit of measure for the scale bars is the number of nucleotide substitutions per site.

For the 44 Neisseria isolates for which WGS were available, phylogenies were generated from the 53 concatenated whole-ribosomal protein gene sequences used in rMLST and for each of the 53 ribosomal protein genes individually. These were compared to identify the single-locus tree that was most congruent with the 53-locus tree in terms of clustering the different taxa. The *rplF* gene phylogeny clustered the sequences consistently with the rMLST tree, and this locus was chosen for further analyses as it was of sufficient length and variability, with conserved flanking regions suitable for primer design. Sanger sequencing of the *rplF* gene using two primers designed from sequences extracted from the WGS data produced a nucleotide fragment of 413 bp, and this determined the length of the *rplF* fragment alleles for the assay. A phylogeny reconstructed from the *rplF* fragment alleles exhibited the same species clusters as the phylogeny produced from the 53 concatenated ribosomal protein gene sequences used in rMLST (Fig. 1).

### *rplF* allele fragment variability.

A total of 27 *rplF* fragment alleles were identified among the set of 44 isolates used to validate the *rplF* assay *in vitro*, which included 10 Neisseria species (see Table S1 in the supplemental material). An examination of the allele sequences from these samples suggested that some isolates had been misidentified. For example, ATCC 19243, originally classified as N. subflava, has been identified as N. mucosa using rMLST. For some isolates, WGS data were unavailable and discrepancies were resolved by examining MLST loci. Of five isolates with *rplF* fragment allele 40, one had been previously identified as N. subflava, whereas four had been identified as N. sicca; however, they had almost identical MLST profiles, differing at only one or two loci and clustered with N. subflava when a phylogeny was reconstructed using concatenated MLST nucleotide sequences (data not shown). With the use of the *rplF* fragment alleles, an isolate identified previously as N. sicca with *rplF* fragment allele 58 was clustered with N. subflava. With the use of concatenated MLST sequences, this isolate also clustered with N. subflava, supporting the species designation identified by the *rplF* assay.

A total of 65 unique alleles of the *rplF* fragment were identified among 926 isolates present in the PubMLST Neisseria database at the time of analysis. Each allele was assigned to a genospecies as described previously (Table 1). N. mucosa, N. sicca, and N. macacae are now considered one species (N. mucosa), as they clustered as one group using rMLST (8). These organisms exhibit either indistinguishable (2) or highly similar 16S rRNA sequences (21). N. flavescens is now considered to be the same species as N. subflava, as these two species were indistinguishable using rMLST (8). The *rplF* fragment alleles were specific for each species group, except for allele 21, which was present in N. mucosa as well as a species previously defined as “Neisseria mucosa var. heidelbergensis” (22), now renamed N. oralis (23). Among WGS data for 804 N. meningitidis and 17 N. gonorrhoeae isolates, there were 6 and 2 unique *rplF* fragment alleles, respectively. The *rplF* fragment alleles from N. polysaccharea and N. meningitidis, the two species most closely related to the type species N. gonorrhoeae (4), were most similar to N. gonorrhoeae allele 7, with 10 and 12 nucleotide differences, respectively. Fixed nucleotide sequence differences were present among all species groups examined, with N. polysaccharea and N. meningitidis alleles having four and seven fixed differences, respectively, from allele 7. Although the sequences from N. polysaccharea and N. meningitidis were similar, there were 15 polymorphisms and 5 fixed differences that differentiated these two species. Compared to allele 7, the *rplF* fragment alleles from the other species of Neisseria were more distantly related, with the allele from a novel Neisseria species (isolate CCUG 21444), originally defined as N. cinerea, having 120 nucleotide differences.

**TABLE 1:** Species associations of *rplF* fragment alleles among 926 Neisseria isolates

Species	No. of isolates	No. of alleles	Allele(s)^*d*^	No. of polymorphic sites	No. of fixed differences
N. gonorrhoeae	17	2	5, 7	1	1
N. polysaccharea	12	3	9, 39, 44	10	4
N. meningitidis	804	6	1, 2, 3, 4, 8, 18	12	7
N. oralis	4	4	21, 26, 36, 68	49	42
N. elongata	4	4	15, 37, 60, 75	50	45
“N. bergeri”^*a*^	1	1	16	63	63
N. lactamica	15	6	6, 32, 33, 34, 51, 52	65	56
N. cinerea	8	5	10, 19, 20, 45, 74	70	51
N. subflava^*b*^	34	14	11, 12, 23, 25, 31, 38, 40, 42, 43, 53, 56, 57, 58, 59	72	49
N. animalis	1	1	76	72	72
N. mucosa^*c*^	16	12	13, 14, 17, 21, 22, 27, 28, 29, 30, 35, 41, 54	76	36
N. dentiae	1	1	47	82	82
N. canis	1	1	48	102	102
N. wadsworthii	1	1	77	102	102
N. weaveri	1	1	24	102	102
N. bacilliformis	4	2	46, 49	115	111
N. shayeganii	1	1	78	116	116
Neisseria sp. (novel)	1	1	50	120	120

a Strain originally defined as N. polysaccharea (20), but rMLST shows that it is a distinct novel species (4) which has yet to be validly published.

b Includes the previous species N. flavescens.

c Includes the previous species N. sicca and N. macacae.

d All alleles are compared to allele 7 from type species N. gonorrhoeae.

### Comparison with 16S rRNA species identification.

**FIG 2:**
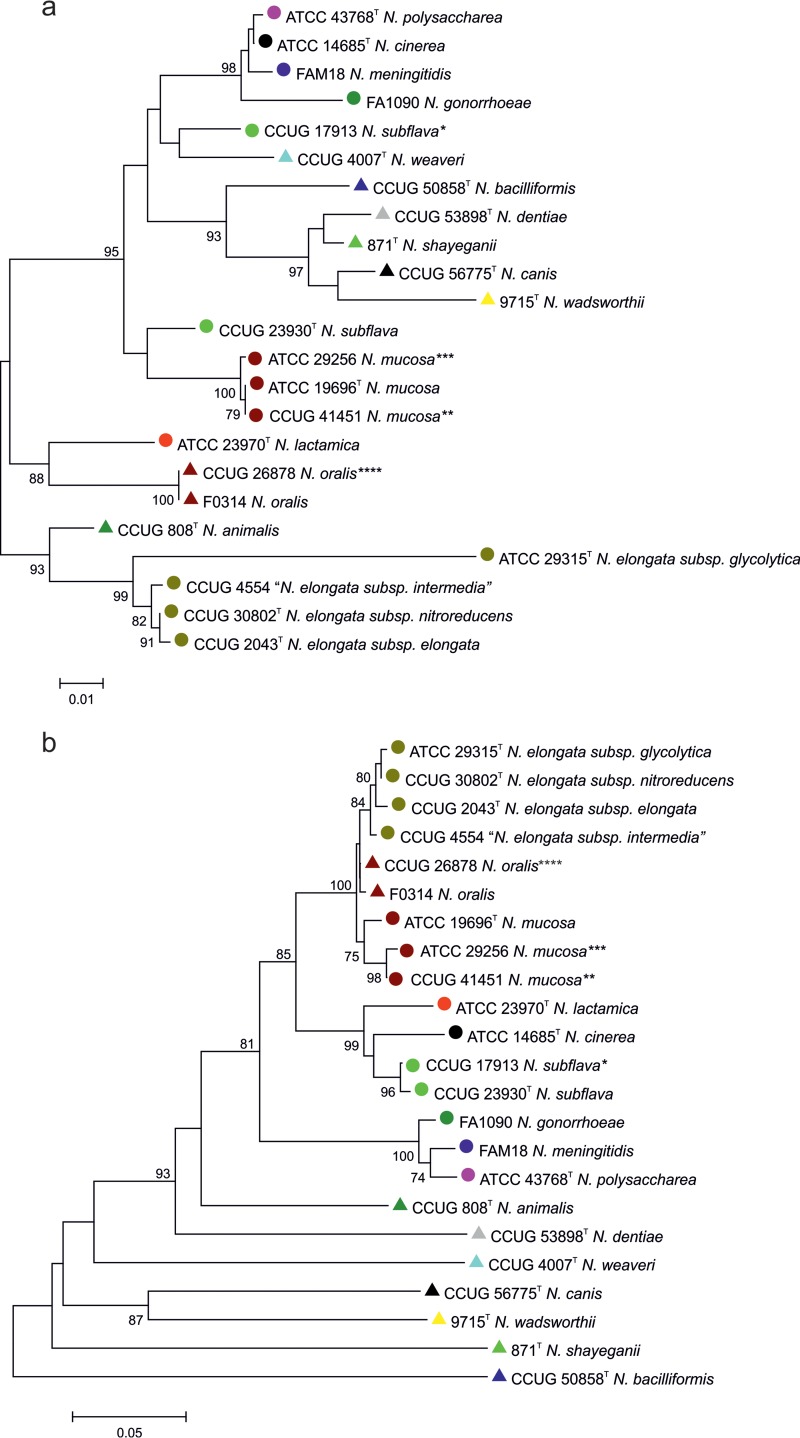
Evolutionary relationships among Neisseria species based on fragments from 16S rRNA and *rplF* genes. (a) 16S rRNA gene fragments. (b) *rplF* gene fragments. ^T^, type strain. Type strains of previous species: *, N. flavescens; **, N. macacae; ***, *N. sicca*; ****, N. mucosa var. heidelbergensis. The percentages of replicate trees in which the associated taxa clustered together in the bootstrap test (1,000 replicates) are shown next to the branches. The unit of measure for the scale bars is the number of nucleotide substitutions per site.

Comparison of a phylogeny reconstructed from the *rplF* fragment alleles from Neisseria type strains with a phylogeny reconstructed using 16S rRNA gene allele fragments (5) demonstrated improved resolution of members of the genus achieved with the *rplF* fragment phylogeny. Species relationships determined using *rplF* fragment alleles were more consistent with rMLST species identification and DNA-DNA hybridization studies (24) than relationships inferred from 16S rRNA gene phylogenies (Fig. 2). The *rplF* fragment allele phylogeny also clustered the more closely related species that are often found in the human oropharynx separately from the more distantly related species that are not associated with humans. A search of the PubMLST Neisseria database also revealed that some 16S rRNA gene sequences are present in both commensals and meningococci. For example, 16S rRNA gene fragment allele 5, originally identified in isolates belonging to the species N. polysaccharea and N. cinerea, including the type strain of N. cinerea, was harbored by three pathogenic serogroup W, meningococcal isolates. Allele 46 has also been found in both an N. polysaccharea isolate and a serogroup B, invasive meningococcus.

### Identifying Neisseria *rplF* fragment alleles using the PubMLST Neisseria database.

To identify a species using an *rplF* fragment, the PubMLST Neisseria database can be queried using the sequence query interface. Users should choose “*rplF* species” and then paste in their nucleotide sequence. If there is an exact match, an *rplF* genospecies designation is returned. If there are polymorphisms present, the closest match is shown and any nucleotide differences are identified and shown in an alignment, which can then be translated. All known *rplF* fragment alleles can be downloaded from the Neisseria locus/sequence definitions database in PubMLST, as can the *rplF* profiles. The Isolate database can also be searched for any related provenance data. In order to assign a new allele, novel *rplF* sequences can be submitted via PubMLST and a curator will then assign a provisional species identity by comparing the percentage identity to known species-specific alleles within the database and reconstructing a phylogeny using all known *rplF* fragment alleles and the novel allele.

## DISCUSSION

The human body hosts a complex microbiota that is important in both health and disease (25). In the case of the genus Neisseria, for example, a variety of species colonize the mouth and oropharynx, with co-colonization providing a reservoir for horizontal genetic exchange (26). While most Neisseria species are harmless commensals, the meningococci and gonococci are important pathogens, and understanding the transition from commensal to pathogen is important in understanding their disease epidemiology (27). Phenotypic characteristics, such as nutritional requirements and biochemical tests, have provided the basis of diagnostic microbiology for many years; however, there are limitations with these methods and the results obtained can be ambiguous, with N. cinerea isolates, for example, being misidentified as gonococci (28, 29). Misidentification of Neisseria can have serious medicolegal consequences (28), as well as distorting the results of epidemiological studies.

Molecular techniques have increasingly replaced phenotypic approaches for characterizing commensal and pathogenic bacteria, with the sequencing of 16S rRNA gene fragments widely employed in diagnostic applications and studies of the microbiome (25, 30, 31). Limitations of this target, due to the similarity of 16S rRNA genes present in different species, are exemplified by Neisseria. For example, there are indistinguishable 16S rRNA gene sequences in N. polysaccharea, N. cinerea, and the meningococci, and some meningococci contain a 16S rRNA gene sequence identical to that found in gonococci (6). Further, public 16S rRNA databases, such as the Human Oral Microbiome database (32) and the EzTaxon-e database (3), can provide misleading results. The closest match to the 16S rRNA gene sequence from N. lactamica 020–06 (33) in both databases is a meningococcal sequence.

A variety of other approaches have been investigated to address this problem, for example, the phylogenetic analysis of the nucleotide sequences of the seven MLST loci, sometimes referred to as multilocus sequence analysis (MLSA) (34). This approach was very effective in distinguishing N. meningitidis, N. gonorrhoeae, and N. lactamica (19) but did not group all members of the genus into species-specific clusters (4). Another method with promise is matrix-assisted laser desorption–ionization time of flight mass spectrometry (MALDI-TOF); however, this method requires optimization, as it has been shown only to separate Neisseria into three groups, *N. meningitis*, N. gonorrhoeae, and other species (35). The availability of rapid and inexpensive whole-genome sequencing and the gene-by-gene approach (36), as implemented in the BIGSdb software (9), has allowed techniques to be developed such as rMLST, which unambiguously identifies species and accurately determines relationships among Neisseria species (4, 7); however, rMLST requires WGS data or the analysis of multiple sequences which, while definitive, is not necessarily feasible or cost-effective for clinical specimens.

A short (413-bp) fragment of the *rplF* gene which encodes the 50S ribosomal protein L6 was found to be a suitable genetic target for rapid differentiation within Neisseria species, as phylogenies reconstructed from *rplF* fragment alleles were consistent with a phylogeny reconstructed from the concatenated sequences of 53 whole-ribosomal protein genes. The *rplF* gene variable region is flanked by conserved regions, a characteristic that enables this fragment to be sequenced on both DNA strands with two primers. Among 65 distinct alleles of this gene fragment identified among 926 isolates, none were shared among commensals and pathogens or between the meningococci and the gonococci, confirming the suitability of the *rplF* fragment assay in differentiating pathogenic and commensal Neisseria species. Only one fragment allele (*rplF* 21) was found in more than one species (N. oralis and N. mucosa), neither of which have been known to cause disease. Although the sequence clusters obtained with the *rplF* fragment alleles were the same as those obtained with concatenated ribosomal protein gene sequences, the phylogeny reconstructed from them was not identical. Consequently, this single genetic target should not be used on its own to define a species or used as a replacement for rMLST. The *rplF* assay is, however, a practical, rapid and inexpensive single-locus tool to differentiate among species within the genus Neisseria which can be combined with additional single-locus tests, such as *porA* sequencing (37) and capsule gene sequencing (38), for example, to confirm meningococcal identity. The assay was specifically developed to identify Neisseria species as part of the MenAfriCar study and has been successfully used to characterize thousands of samples from heat-killed cell suspensions, assisting in determining the impact of serogroup A polysaccharide conjugate vaccines on meningococcal carriage (1, 39).

The *rplF* fragment allele sequences and associated metadata are stored in the PubMLST Neisseria database. It is curated and continually updated, providing an extensive library of genomes and DNA sequences along with the tools to analyze these data. Although the majority of the isolates are meningococci, it contains a number of representative strains from most species, including culture collection strains, as well as isolates from population studies, which can be used to query sequences to provide a species identity. While the *rplF* gene fragment assay is specific for Neisseria, the general approach can be adapted to identify other bacterial species, as the *rp* genes are universal (7). However, the *rplF* gene fragment assay has not, at the time of this writing, been adapted to identify species within other genera. In addition to species identification, ribosomal genes have potential applications in the investigation of noncultured samples and in metagenomic studies, where resolution finer than that provided by the 16S rRNA gene is required. (20).

## Supplementary Material

Supplemental material

## References

[1] Doumagoum Moto D Gami JP Gamougam K Naibei N Mbainadji L Narbé M Toralta J Kodbesse B Ngadoua C Coldiron M Ferman F Page A-L Djingarey M Hugonnet S Harrison OB Rebbetts LS Tekletsion Y Watkins ER Caugant DA Chandramohan D Hassan-King M Manigart O Nascimento M Woukeu A Trotter C Stuart JM Maiden MCJ Greenwood B 2013 Effect of a serogroup A meningococcal conjugate vaccine (PsA-TT) on serogroup A meningococcal meningitis and carriage in Chad: a community study [corrected]. Lancet 83:40–47. 10.1016/S0140-6736(13)61612-8 PMC3898950 24035220

[2] Harmsen D Singer C Rothganger J Tonjum T de Hoog GS Shah H Albert J Frosch M 2001 Diagnostics of Neisseriaceae and *Moraxellaceae* by ribosomal DNA sequencing: ribosomal differentiation of medical microorganisms. J. Clin. Microbiol. 39:936–942. 10.1128/JCM.39.3.936-942.2001 11230407 PMC87853

[3] Kim OS Cho YJ Lee K Yoon SH Kim M Na H Park SC Jeon YS Lee JH Yi H Won S Chun J 2012 Introducing EzTaxon-e: a prokaryotic 16S rRNA gene sequence database with phylotypes that represent uncultured species. Int. J. Syst. Evol. Microbiol. 62:716–721. 10.1099/ijs.0.038075-0 22140171

[4] Bennett JS Jolley KA Earle SG Corton C Bentley SD Parkhill J Maiden MC 2012 A genomic approach to bacterial taxonomy: an examination and proposed reclassification of species within the genus Neisseria. Microbiology 158:1570–1580. 10.1099/mic.0.056077-0 22422752 PMC3541776

[5] Harmsen D Rothganger J Frosch M Albert J 2002 RIDOM: ribosomal differentiation of medical microorganisms database. Nucleic Acids Res. 30:416–417. 10.1093/nar/30.1.416 11752353 PMC99060

[6] Walcher M Skvoretz R Montgomery-Fullerton M Jonas V Brentano S 2013 Description of an unusual N. meningitidis isolate containing and expressing N. gonorrhoeae-specific 16S rRNA gene sequences. J. Clin. Microbiol. 51:3199–3206. 10.1128/JCM.00309-13 23863567 PMC3811666

[7] Jolley KA Bliss CM Bennett JS Bratcher HB Brehony CM Colles FM Wimalarathna HM Harrison OB Sheppard SK Cody AJ Maiden MC 2012 Ribosomal multilocus sequence typing: universal characterization of bacteria from domain to strain. Microbiology 158:1005–1015. 10.1099/mic.0.055459-0 22282518 PMC3492749

[8] Bennett JS Jolley KA Maiden MC 2013 Genome sequence analyses show that Neisseria oralis is the same species as “Neisseria mucosa var. heidelbergensis.” Int. J. Syst. Evol. Microbiol. 63:3920–3926. 10.1099/ijs.0.052431-0 24097834 PMC3799226

[9] Jolley KA Maiden MC 2010 BIGSdb: scalable analysis of bacterial genome variation at the population level. BMC Bioinformatics 11:595. 10.1186/1471-2105-11-595 21143983 PMC3004885

[10] Edgar RC 2004 MUSCLE: multiple sequence alignment with high accuracy and high throughput. Nucleic Acids Res. 32:1792–1797. 10.1093/nar/gkh340 15034147 PMC390337

[11] Tamura K Peterson D Peterson N Stecher G Nei M Kumar S 2011 MEGA5: molecular evolutionary genetics analysis using maximum likelihood, evolutionary distance, and maximum parsimony methods. Mol. Biol. Evol. 28:2731–2739. 10.1093/molbev/msr121 21546353 PMC3203626

[12] Kimura M 1980 A simple method for estimating evolutionary rates of base substitutions through comparative studies of nucleotide sequences. J. Mol. Evol. 16:111–120. 10.1007/BF01731581 7463489

[13] Felsenstein J 1985 Confidence limits on phylogenies: an approach using the bootstrap. Evolution 39:783–791. 10.2307/2408678 28561359

[14] Librado P Rozas J 2009 DnaSP v5: a software for comprehensive analysis of DNA polymorphism data. Bioinformatics 25:1451–1452. 10.1093/bioinformatics/btp187 19346325

[15] Ye J Coulouris G Zaretskaya I Cutcutache I Rozen S Madden TL 2012 Primer-BLAST: a tool to design target-specific primers for polymerase chain reaction. BMC Bioinformatics 13:134. 10.1186/1471-2105-13-134 22708584 PMC3412702

[16] Embley TM 1991 The linear PCR reaction: a simple and robust method for sequencing amplified rRNA genes. Lett. Appl. Microbiol. 13:171–174. 10.1111/j.1472-765X.1991.tb00600.x 1370053

[17] Staden R 1996 The Staden sequence analysis package. Mol. Biotechnol. 5:233–241. 10.1007/BF02900361 8837029

[18] Altschul SF Gish W Miller W Myers EW Lipman DJ 1990 Basic local alignment search tool. J. Mol. Biol. 215:403–410. 10.1006/jmbi.1990.9999 2231712

[19] Bennett JS Jolley KA Sparling PF Saunders NJ Hart CA Feavers IM Maiden MC 2007 Species status of Neisseria gonorrhoeae: evolutionary and epidemiological inferences from MLST. BMC Biol. 5:35. 10.1186/1741-7007-5-35 17825091 PMC2031879

[20] Berger U 1985 First isolation of *Neisseria polysacchareae* species nova in the Federal Republic of Germany. Eur. J. Clin. Microbiol. 4:431–433. 10.1007/BF02148705 4043067

[21] Tonjum T 2005 Genus I. Neisseria, p 777–798 *In* Garrity GM Brenner DJ Krieg NR Staley JR (ed), Bergey's manual of systematic bacteriology, 2nd ed, vol 2 Springer-Verlag, New York, NY

[22] Berger U 1971 Neisseria mucosa var. heidelbergensis. Z Med. Mikrobiol. Immunol. 156:154–158 (In German.) 10.1007/BF02124646 5554812

[23] Wolfgang WJ Passaretti TV Jose R Cole J Coorevits A Carpenter AN Jose S Van Landschoot A Izard J Kohlerschmidt DJ Vandamme P Dewhirst FE Fisher MA Musser KA 2013 Neisseria oralis sp. nov. isolated from healthy gingival plaque and clinical samples. Int. J. Syst. Evol. Microbiol. 63(Pt 4):1323–1328. 10.1099/ijs.0.041731-0 22798652 PMC3709538

[24] Guibourdenche M Popoff MY Riou JY 1986 Deoxyribonucleic acid relatedness among Neisseria gonorrhoeae, N. meningitidis, N. lactamica, N. cinerea and “Neisseria polysaccharea.” Ann. Inst. Pasteur. Microbiol. 137B:177–185 10.1016/s0769-2609(86)80106-5 3120761

[25] Dewhirst FE Chen T Izard J Paster BJ Tanner AC Yu WH Lakshmanan A Wade WG 2010 The human oral microbiome. J. Bacteriol. 192:1422–1431. 10.1128/JB.00542-10 PMC2944498 20656903

[26] Kong Y Ma JH Warren K Tsang RS Low DE Jamieson FB Alexander DC Hao W 2013 Homologous recombination drives both sequence diversity and gene content variation in Neisseria meningitidis. Genome Biol. Evol. 5:1611–1627. 10.1093/gbe/evt116 23902748 PMC3787668

[27] Maiden MC 2008 Population genomics: diversity and virulence in the Neisseria. Curr. Opin. Microbiol. 11:467–471. 10.1016/j.mib.2008.09.002 18822386 PMC2612085

[28] Dossett JH Appelbaum PC Knapp JS Totten PA 1985 Proctitis associated with Neisseria cinerea misidentified as Neisseria gonorrhoeae in a child. J. Clin. Microbiol. 21:575–577 3921562 10.1128/jcm.21.4.575-577.1985 PMC271722

[29] Knapp JS Totten PA Mulks MH Minshew BH 1984 Characterization of Neisseria cinerea, a nonpathogenic species isolated on Martin-Lewis medium selective for pathogenic Neisseria spp. J. Clin. Microbiol. 19:63–67 6361062 10.1128/jcm.19.1.63-67.1984 PMC270980

[30] Faust K Sathirapongsasuti JF Izard J Segata N Gevers D Raes J Huttenhower C 2012 Microbial co-occurrence relationships in the human microbiome. PLoS Comput. Biol. 8:e1002606. 10.1371/journal.pcbi.1002606 22807668 PMC3395616

[31] Huse SM Ye Y Zhou Y Fodor AA 2012 A core human microbiome as viewed through 16S rRNA sequence clusters. PLoS One 7:e34242. 10.1371/journal.pone.0034242 22719824 PMC3374614

[32] Chen T Yu WH Izard J Baranova OV Lakshmanan A Dewhirst FE 2010 The Human Oral Microbiome Database: a web accessible resource for investigating oral microbe taxonomic and genomic information. Database 2010:baq013. 10.1093/database/baq013 20624719 PMC2911848

[33] Bennett JS Bentley SD Vernikos GS Quail MA Cherevach I White B Parkhill J Maiden MCJ 2010 Independent evolution of the core and accessory gene sets in the genus Neisseria: insights gained from the genome of Neisseria lactamica isolate 020–06. BMC Genomics 11:652. 10.1186/1471-2164-11-652 21092259 PMC3091772

[34] Gevers D Cohan FM Lawrence JG Spratt BG Coenye T Feil EJ Stackebrandt E Van de Peer Y Vandamme P Thompson FL Swings J 2005 Opinion: re-evaluating prokaryotic species. Nat. Rev. Microbiol. 3:733–739. 10.1038/nrmicro1236 16138101

[35] Ilina EN Borovskaya AD Malakhova MM Vereshchagin VA Kubanova AA Kruglov AN Svistunova TS Gazarian AO Maier T Kostrzewa M Govorun VM 2009 Direct bacterial profiling by matrix-assisted laser desorption-ionization time-of-flight mass spectrometry for identification of pathogenic Neisseria. J. Mol. Diagn. 11:75–86. 10.2353/jmoldx.2009.080079 19095774 PMC2607569

[36] Maiden MC van Rensburg MJ Bray JE Earle SG Ford SA Jolley KA McCarthy ND 2013 MLST revisited: the gene-by-gene approach to bacterial genomics. Nat. Rev. Microbiol. 11:728–736. 10.1038/nrmicro3093 23979428 PMC3980634

[37] Russell JE Jolley KA Feavers IM Maiden MC Suker J 2004 PorA variable regions of Neisseria meningitidis. Emerg. Infect. Dis. 10:674–678. 10.3201/eid1004.030247 15200858 PMC3323080

[38] Harrison OB Claus H Jiang Y Bennett JS Bratcher HB Jolley KA Corton C Care R Poolman JT Zollinger WD Frasch CE Stephens DS Feavers I Frosch M Parkhill J Vogel U Quail MA Bentley SD Maiden MCJ 2013 Description and nomenclature of Neisseria meningitidis capsule locus. Emerg. Infect. Dis. 19:566–573. 10.3201/eid1904.111799 23628376 PMC3647402

[39] Ali O Aseffa A Bedru A Lemma T Moti T Workhu A Guebre Xabher H Yamuah L Boukary RM Collard JM Dano ID Habiboulaye I Issaka B Jusot JF Ousmane S Rabe I Gami JP Gamougam K Kodbesse B Naibei N Ngadoua C Mbainadji L Moto DD Narbe M Toralta J Berthe A Keita M Diallo K Onwuchekwa U Sow SO Tamboura B Traore A Toure A Amodu M Beida O Gadzama G Omotara B Sambo Z Yahya S Chandramohan D Greenwood B Hassan-King M Manigart O Nascimento M Stuart J Woukeu A Bai X Borrow R Findlow H Avalo S Bassene H Diallo A Dieng M Doucoure S Gomis JF Ndiaye A Sokhna C Trape JF Akalifa B Forgor A Hodgson A Osei I Quaye S Williams J Wontuo P Basta N Irving T Trotter C Bennett J Hill D Harrison O Rebbetts L Maiden M Tekletsion Y Watkins E 2013 Meningococcal carriage in the African meningitis belt. Trop. Med. Int. Health 18:968–978. 10.1111/tmi.12125 23682910 PMC3950817

