# Supplementary material for: Identifying Neisseria Species by Use of the 50S Ribosomal Protein L6 (rplF) Gene
Source: J Clin Microbiol. 2014 May;52(5):1375–81. doi: 10.1128/JCM.03529-13 (PMC3993661; doi:10.1128/JCM.03529-13)
Supplement: Supplemental material [file supp_52_5_1375__index.html]

Identifying Neisseria Species by Use of the 50S Ribosomal Protein L6 (rplF) Gene — Supplemental material 

# Identifying Neisseria Species by Use of the 50S Ribosomal Protein L6 (*rplF*) Gene

## Supplemental material

**Files in this Data Supplement:**

- Supplemental file 1 -

  Table S1 (Isolates used to validate the *rplF* assay *in vitro*)

  XLSX, 14K
- Supplemental file 2 -

  Table S2 (All isolates used in the study)

  XLSX, 98K
